# Integrating Molecular Perspectives: Strategies for Comprehensive Multi-Omics Integrative Data Analysis and Machine Learning Applications in Transcriptomics, Proteomics, and Metabolomics

**DOI:** 10.3390/biology13110848

**Published:** 2024-10-22

**Authors:** Pedro H. Godoy Sanches, Nicolly Clemente de Melo, Andreia M. Porcari, Lucas Miguel de Carvalho

**Affiliations:** 1 MS4Life Laboratory of Mass Spectrometry, Health Sciences Postgraduate Program, São Francisco University, Bragança Paulista 12916-900, SP, Brazil; 2 Graduate Program in Biomedicine, São Francisco University, Bragança Paulista 12916-900, SP, Brazil; 3 Post Graduate Program in Health Sciences, São Francisco University, Bragança Paulista 12916-900, SP, Brazil

**Keywords:** omics data, transcriptomics, metabolomics, proteomics, omics integration, multi-omics

## Abstract

**Simple Summary:**

Recent high-throughput technologies such as transcriptomics, proteomics, and metabolomics have allowed progress in understanding biological systems at different levels of detail. Even so, it is necessary to integrate multiple omics data sets to achieve a comprehensive understanding of the subject under study. In this article, we review the methods used for integrating transcriptomics, proteomics, and metabolomics data and summarize them in three approaches: combined omics integration, correlation-based integration strategies, and machine learning integrative approaches. Our goal is to showcase the uses and limitations of each approach, allowing researchers to choose the more appropriate tool for each scenario to extract a comprehensive view of a biological system.

**Abstract:**

With the advent of high-throughput technologies, the field of omics has made significant strides in characterizing biological systems at various levels of complexity. Transcriptomics, proteomics, and metabolomics are the three most widely used omics technologies, each providing unique insights into different layers of a biological system. However, analyzing each omics data set separately may not provide a comprehensive understanding of the subject under study. Therefore, integrating multi-omics data has become increasingly important in bioinformatics research. In this article, we review strategies for integrating transcriptomics, proteomics, and metabolomics data, including co-expression analysis, metabolite–gene networks, constraint-based models, pathway enrichment analysis, and interactome analysis. We discuss combined omics integration approaches, correlation-based strategies, and machine learning techniques that utilize one or more types of omics data. By presenting these methods, we aim to provide researchers with a better understanding of how to integrate omics data to gain a more comprehensive view of a biological system, facilitating the identification of complex patterns and interactions that might be missed by single-omics analyses.

## 1. Introduction

Omics data result from the use of large-scale instruments used in biology. They can contain measurements of different biomolecules, their functions, and interactions [1]. They have become an essential tool in modern biology and biomedicine [2,3]. Transcriptomics, proteomics, and metabolomics are three major omics fields that provide different types of biological information.

As an indirect measure of DNA activity [4], transcriptomics measures the expression levels of a set of RNA transcripts (mRNA, non-coding RNA, etc.) in a cell or tissue, i.e., the transcriptome. Produced according to the instructions from mRNA, proteins and enzymes, typically > 2 kDa, are the functional products of genes and play several roles in cellular processes [5], being the macromolecule responsible for direct interactions among cells and tissues, besides maintaining the cellular structure [6]. Proteomics then focuses on the identification and quantification of a set of proteins, the *proteome* [7]. Smaller molecules (≤1.5 kDa), referred to as metabolites, are intermediate or end products of metabolic reactions and regulators of metabolism [8], but are not analyzed with the instrumental methodologies used in proteomics. Metabolomics comprehensively analyzes these molecules, trying to describe and quantify the molecular composition of a sample (the metabolome). Additionally, a big branch of metabolomics is the study of the lipidic composition of a sample, its lipidome, and for that, the “Lipidomics” term is used [8]. By indirectly measuring how a gene is acting, transcriptomics covers the upstream processes of metabolism, while proteomics is the intermediate step, defining protein structure and biocommunication. Metabolomics focuses on the regulators and the ultimate mediators of a metabolic process (usually smaller molecules not more than 1.5 kDa) [9]. Together, these omics technologies offer a comprehensive and streamlined view of biological processes.

Integrating multiple omics data sets is a challenging but necessary task to fully understand complex biological systems. Data integration can provide novel biological insights and reveal previously unknown relationships between different molecular components. Moreover, it can help identify biomarkers and therapeutic targets for various diseases. Several methods have been developed for integrating omics data, including correlation-based approaches, machine learning algorithms, and network-based analyses [4,10,11,12]

In this article, we will review and discuss different methods for integrating transcriptomics, proteomics, and metabolomics data. We will discuss the strengths and limitations of each method and provide examples of their applications in various biological contexts. Also, we will cite strategies and articles that use these omics in machine learning-based studies. By doing so, we hope to contribute to the development of effective strategies for omics data integration and pave the way for new discoveries in biomedical and biotechnology research.

## 2. Methods for Integrating Multi-Omics Data

Integrating omics data from several domains is critical for gaining complete knowledge of biological systems. To uncover critical regulatory pathways and networks, transcriptomics data can be combined with proteomics or metabolomics data. Many methodologies for integrating transcriptomics and proteomics data have been developed, including correlation-based approaches and pathway and co-expression analysis. Merging proteomics data with metabolomics data is also a potential strategy for biomarker development and disease diagnosis, since it can uncover alterations in metabolic pathways linked to disease states. Co-expression analysis and network-based techniques have been utilized successfully in the integration of transcriptomics and metabolomics. Overall, integrating omics data can be a significant tool for deciphering complicated biological systems and discovering novel treatment targets [3,10,12,13,14,15,16].

**Figure 1:**
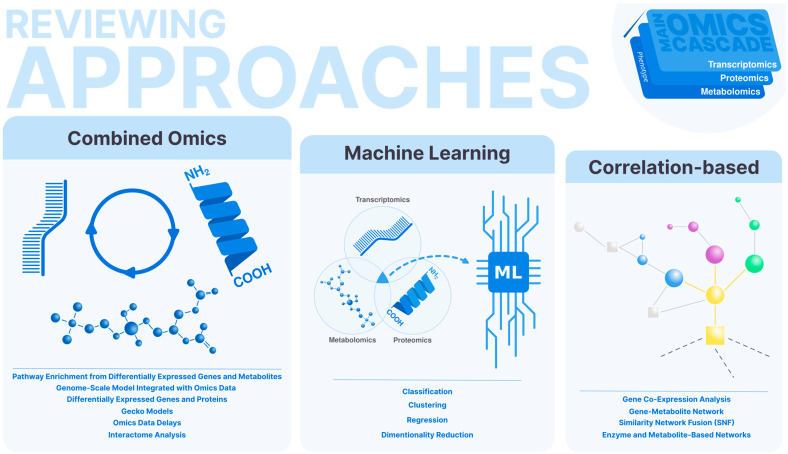
Strategies for integrating omics data. Methods are based on correlation-based approaches, which identify associations between different types of data; machine learning algorithms, which can predict outcomes and identify patterns across data sets; and combined individual approaches, which map the interactions and relationships between molecular components.

We divide the methods of omics integration into three major approaches: combined omics integration, correlation-based integration strategies, and machine learning integrative approaches (Figure 1). Combined omics integration approaches attempt to explain what occurs within each type of omics data in an integrated manner, generating independent data sets. Correlation-based strategies apply correlations between the generated omics data and create data structures to represent these relationships, such as networks. Finally, machine learning strategies utilize one or more types of omics data, potentially incorporating additional information inherent to these data sets, to comprehensively understand responses at the classification and regression levels, particularly in relation to diseases. These methods enable a comprehensive view of biological systems, facilitating the identification of complex patterns and interactions that might be missed by single-omics analyses. By leveraging these integrative approaches, researchers can achieve deeper insights into the molecular mechanisms underlying health and disease, ultimately aiding in the discovery of novel biomarkers and therapeutic targets.

In this article, we focus on non-approximate strategies for cell-to-cell communication, such as single-cell RNA-seq (scRNA-seq), due to their level of resolution and ability to detect communication between individual cells. Bulk analysis assumes that cells are identical and can model the exchange between cells and the environment [17]. However, we will provide excellent recent reviews that present strategies for integrating multi-omics data from single-cell data [17,18,19,20,21,22,23,24,25].

### 2.1. Correlation-Based Methods

Correlation-based strategies involve applying statistical correlations between different types of generated omics data to uncover and quantify relationships between various molecular components. These methods, summarized in Table 1, then create data structures, such as networks, to visually and analytically represent these relationships. By mapping out these correlations, researchers can identify patterns of co-expression, co-regulation, and functional interactions that occur across different omics layers. This approach allows for the detection of complex interdependencies and the construction of interaction networks that highlight key molecules and pathways involved in biological processes.

#### 2.1.1. Gene Co-Expression Analysis Integrated with Metabolomics Data

Co-expression analysis is a powerful approach for identifying genes with the same expression pattern that may participate in the same biological pathways or have the same biological function [26,27]. One strategy for integrating transcriptomics and metabolomics data is to perform a co-expression analysis on transcriptomics data and identify gene modules that are co-expressed. These modules can then be linked to metabolites identified from metabolomics data to identify metabolic pathways that are co-regulated with the identified gene modules [28,29,30,31,32,33,34].

To further understand the relationship between co-expressed genes and metabolites, the correlation between metabolite intensity patterns and the eigengenes of each co-expression module can be calculated. Eigengenes are representative expression profiles for each module that summarize the overall expression pattern of the genes within the module. By correlating these eigengenes with metabolite intensity patterns, it is possible to identify which metabolites are most strongly associated with each co-expression module [35,36,37,38,39]. Additionally, you can use the normalized metabolomics data directly in Weighted Correlation Network Analysis (WGCNA) [27], conducting a module–sample relationship analysis (in this case, module–metabolite relationship), and identify relationships between module eigengenes and metabolite intensity.

This approach can provide important insights into the regulation of metabolic pathways and the formation of specific metabolites. For example, if a particular co-expression module is strongly correlated with the production of a specific metabolite, it suggests that the genes within the module are involved in regulating the metabolic pathway leading to that metabolite. By combining transcriptomics and metabolomics data, it is possible to identify key genes and metabolic pathways involved in specific biological processes or disease states and to develop targeted interventions to modulate these processes, taking into account that the biological conditions of both omics analyses must be the same.

#### 2.1.2. Gene–Metabolite Network

A gene–metabolite network is a visualization of the interactions between genes and metabolites in a biological system. Generating and analyzing these networks involves collecting gene expression and metabolite abundance data, integrating the data, constructing the network, analyzing it, and interpreting the results. Gene–metabolite networks can help identify key regulatory nodes and pathways that are involved in metabolic processes and can be used to generate hypotheses about the underlying biology.

To generate a gene–metabolite network, researchers must first collect gene expression and metabolite abundance data from the same biological samples. These data are then integrated using Pearson correlation coefficient (PCC) analysis or other statistical methods to identify genes and metabolites that are co-regulated or co-expressed [40,41,42]. For example, Nikiforova et al., 2005, exhibited a systematic procedure to construct a gene–metabolite network based on the profiles of transcripts and metabolites [43]. Also, gene–metabolite networks are constructed using visualization software, such as Cytoscape [44] or igraph [45], with genes and metabolites represented as nodes in the network and connected with edges that represent the strength and direction of their interactions.

Once the network is constructed, it can be analyzed using network analysis tools to identify key regulatory nodes and pathways that are involved in metabolic processes [46,47,48,49,50]. Furthermore, a gene–metabolite network could be constructed with genes and metabolites specifically deregulated, to focus on the process that is modulated by each biological condition. Validation and interpretation of the network can then be performed by comparing it to known metabolic pathways and regulatory networks and using pathway analysis, gene ontology analysis, and functional enrichment analysis to identify enriched pathways and processes. Afterwards, you may select genes present in metabolic pathways or biological processes and obtain the mRNA levels of these genes by real-time qRT-PCR.

One interesting way of constructing a metabolite–gene network is based on the corto package developed by Mercatelli D. et al. (2020) [51]. First, the metabolite and gene data are combined into a single matrix, with only the metabolites designated as “centroids” or hubs in the resulting co-occurrence network. To determine significant edges in the network, you may set the minimum Pearson correlation coefficient *p*-value to 0.05 or a chosen cut-off. To test the significance of each edge in the network, it is necessary to conduct 100 or more bootstraps. The R code snippet below describes the implementation of this method, with “full_matrix” as the input matrix, and “metabolites” as a vector containing the metabolite names, as proposed by Cavicchioli M.V. et al. (2022) [52].

#### 2.1.3. Similarity Network Fusion (SNF)

Similarity Network Fusion (SNF) [53] is a computational approach for integrating various data types [54,55]. In essence, SNF merges diverse measurements, such as mRNA expression, protein abundance, miRNA expression, metabolomics, clinical data, questionnaires, and image data, among others, for a given set of samples, like patients. Essentially, it starts by creating a sample similarity network for each data type and then combines these networks iteratively using a unique network fusion technique. By operating in the sample network space, SNF effectively circumvents issues related to different scales, collection biases, and noise in various data types.

For example, let us say that we have transcriptomics data from N patients (treated vs. control) and metabolomics data from the same patients. The SNF algorithm constructs a similarity network for each omics data set separately, where each node represents a patient, and the edge intensity connecting two patients indicates the level of association based on that omics data set. Subsequently, both networks are merged, and edges with high associations in each omics network are highlighted. Clustering can be performed to identify phenotypic associations among patients and cancer types, for instance. Furthermore, patient information (such as weight, survival time, among others) can be added to the nodes to enhance information visualization. Looking at the final network and examining each edge and its source (whether it is supported by one or more omics datasets), we can see how each omics dataset supports each group of patients. This allows for comparisons between different diseases. We highlight that there are limitations in the SNF method, such as dependence on pre-processing, noise in the input data, and computational complexity.

#### 2.1.4. Enzyme and Metabolite-Based Network

In this approach, we utilize the available metabolomics and proteomics data to identify a network of protein–metabolite or enzyme–metabolite interactions using genome-scale models or pathways databases and then combine it with omics data such as fold changes to visualize the enriched pathways. There are two main strategies to define such networks, based on either proteomics or metabolomics data in the first step.

Let us start with the proteomics-based strategy. First, we identify the reactions in which the identified proteins participate in the genome-scale model, which can be in the GPR in AND or OR operator. Then, we determine the metabolites that are consumed (met-c) and produced (met-p) in those reactions. This information is used to construct a protein–metabolite network, where the protein is derived from the identification of the GPR, and the metabolites are the reactions that the protein is involved in (met-c and met-p). Additional details such as compartments, reactions ID, and fold changes can be included to enhance the network.

The second strategy relies on metabolomics data, which require standardization of the metabolite names or IDs in the database. Using the identified metabolites, along with the genome-scale model, we identify all the reactions in which those metabolites participate and annotate the proteins belonging to the GPR, in AND or OR operator. Then, we assemble the protein–metabolite network using this interaction information.

Both strategies are based on omics data with the addition of genome-scale models as the foundation for assembling the network structure. Although the associations from the Kyoto Encyclopedia of Genes and Genomes (KEGG) can be used directly, we expand to a whole genome-scale model. The first strategy yields a larger network due to the greater number of identifications that proteomics data provide compared to metabolomics data.

MetaBridge [56] is a powerful web-based tool that aims to integrate metabolomics with proteomics data. The tool achieves this by utilizing data from two key databases: the MetaCyc metabolic pathway database [57] and KEGG. MetaBridge maps metabolite compounds to interacting upstream or downstream enzymes in enzymatic reactions and metabolic pathways and generates a list of enzymes that can be integrated with proteomics or transcriptomics data using protein–protein interaction (PPI) networks. The resulting PPI network can be used for integrative multi-omics analyses, allowing users to identify key proteins and pathways that are differentially regulated across the data sets and also integrate the fold change per protein and select specific submodules from it. By providing a user-friendly interface and detailed protocols, MetaBridge makes it easy to perform integrative multi-omics analyses and gain insights into complex biological systems.

### 2.2. Combined Omics Approaches

Combined omics integration approaches, as summarized in Table 2, seek to explain the phenomena occurring within each type of omics data through a comprehensive and integrated framework. These methods generate independent data sets for each omics layer, such as transcriptomics, proteomics, and metabolomics, and then combine them to provide a full view of a biological system. This integrated analysis can reveal insights that would not be apparent when examining each omics data set in isolation, thus enabling the identification of novel interactions, pathways, and regulatory mechanisms that drive biological functions and disease states. Here, we will discuss different strategies and the necessary care for integrating omics data.

#### 2.2.1. Pathway Enrichment from Differentially Expressed Genes and Metabolites

One strategy for integrating transcriptomics and metabolomics data is to perform KEGG enrichment analysis on differentially expressed genes (DEGs) identified from transcriptomics data and then link the enriched pathways to the metabolites identified from metabolomics data. This approach can reveal the metabolic pathways that are most affected by changes in gene expression and provide insights into the underlying biological mechanisms. Additionally, fold-change information from DEGs can be integrated with metabolomics data to identify metabolites that are significantly changed in abundance and may play a key role in the observed changes in gene expression.

DEG pathway enrichment may be performed using the clusterProfiler [58] or GSEApy [59] packages, for example. One way to convert a gene name to Entrez Gene ID, which provides unique integer identifiers for genes and other loci, or KEGG ID to perform the enrichment through the clusterProfiler is through the genekitr package using the *transId()* function with the argument *transTo = “entrez”*. The MetaboAnalyst platform [60] may be used to enhance metabolic pathways with identified metabolites. Furthermore, information from gene expression, such as fold changes, and metabolite intensity analysis can be integrated into metabolic pathway figures using the Pathview package [61].

Additionally, it is possible to analyze the correlation between the abundance of metabolites and the expression of genes or transcripts across various biological conditions using integrated pathway analysis. To perform this analysis, it is necessary to include multiple time points or a large number of biological conditions. The association cut-off for this analysis is based on a *p*-value < α and Pearson coefficients > *β*, which can be plotted to determine the extent to which relevant metabolites are correlated with relevant mRNA transcripts. To carry out this analysis, Multi-Omics Factor Analysis (MOFA) [62] is a suitable approach for determining the degree to which changes in metabolite abundance and mRNA expression variables are related [63,64,65]. In addition, correlation analysis can be applied to determine the degree of relationship between metabolite intensities/concentrations and gene expression, but this must be carried out with normalized (post-processing) data. Data normalization in the post-processing of metabolomics data is usually provided, especially if the statistical analyses are conducted in MetaboAnalyst.

Also, if a multivariate analysis is chosen, such as Principal Component Analysis (PCA), it is important to understand the factors that can lead to failure of the analysis of variance. We point out that data integration through concatenation can become complex when the data sets to be merged differ significantly in size. Not only do metabolomics and transcriptomics data sets differ significantly in size, but they are also generated using vastly different technologies [66]. This means that the data sets have distinct structures, unique patterns of expected values, dissimilar distributions of underlying noise, and varying levels of variance. With that in mind, the best proposal is to carry out a PCA biplot for the metabolites and another for the genes, identify the most correlated metabolites and genes either with each biological condition or with each principal component (PC1 or PC2), and then associate this result with the pathway that both belong to.

#### 2.2.2. Integrating Genome-Scale Models with Metabolomics and Transcriptomics Data

Integrating transcriptomics data, genome-scale models (GEMs), and metabolomics data can provide a comprehensive understanding of cellular metabolism and its regulation [67]. One approach to integrating these data types is to generate condition-specific models (CSMs) that incorporate the transcriptomics data and use them to simulate metabolic fluxes in different situations [68,69,70,71,72]. Here, we describe a strategy for generating CSMs using GEMs and transcriptomics data and then integrating these models with metabolomics data.

The first step in this strategy is to generate a GEM that represents the metabolic network of the organism of interest. The GEM should include all the reactions and metabolites involved in cellular metabolism and should be curated and validated using experimental data. Once the GEM is generated, it can be used to simulate metabolic fluxes under different conditions.

Next, transcriptomics data can be used to generate CSMs that incorporate the expression levels of genes under different conditions. This can be achieved using constraint-based modeling techniques such as flux balance analysis (FBA) [73] or parsimonious FBA (pFBA) [74]. CSMs can be generated by constraining the fluxes through the reactions in the GEM based on the expression levels of the corresponding genes. MEWpy [75] is a package that covers a wide range of metabolic and regulatory modeling approaches, as well as phenotype simulation and Computational Strain Optimization (CSO) algorithms. This makes it a useful tool for generating transcriptome-based simulations with FBA or pFBA.

Once the CSMs are generated, they can be used to simulate metabolic fluxes in different conditions and predict the metabolic phenotypes of a cell. However, to fully understand the regulation of cellular metabolism, it is important to also integrate metabolomics data into the models.

One way to integrate metabolomics data into CSMs is to use the former to constrain the fluxes through the exchange reactions that correspond to the measured metabolites. It is essential to normalize the exchange reactions based on metabolite concentrations in a metabolic model. For example, if an organism cannot consume the entire concentration of a metabolite in 24 h, you can estimate the upper exchange flux as follows: [Concentration]/(1 gDW × 24 h). Moreover, one possibility is using linear programming techniques to find the flux distributions that are consistent with both the transcriptomics and the metabolomics data, and this approximation is used to insert the exchange reaction fluxes. We highlight that there are limitations in the method, such as noise and data variability, differences in temporal scales between the omics data, incomplete annotations in the metabolic model, and limitations in experimental validation.

#### 2.2.3. Gecko Models

In recent years, the integration of omics data into genome-scale metabolic models (GEMs) has become a powerful approach for exploring the relationship between genotype and phenotype for different organisms [76,77,78]. GEMs allow for the prediction of metabolic behavior and can be used to design experiments and engineer biological systems. One such tool that has enabled the integration of proteomics data into GEMs is the GECKO toolbox [79]. GECKO models take into account enzyme and proteomics constraints to study phenotypes that are constrained by protein limitations [80,81,82,83]. With the GECKO toolbox, it is possible to generate enzyme-constrained models (ecModels) for a variety of organisms, including budding yeasts such as *Saccharomyces cerevisiae* and humans, as well as build your own model. These models can be used to study the long-term adaptation of organisms to stress factors and nutrient-limited conditions.

The GECKO models simplify the process of limiting the metabolic fluxes in any GEM that contains enzymatic data, reducing the variability of constraint-based modeling results and improving predictions. This approach is executed by representing enzymes as entities with limited capacities in the corresponding reactions in the model, thereby extending the genome-scale modeling. In traditional genome-scale modeling, a stoichiometric matrix is defined that represents the whole metabolism, with columns indicating each reaction’s stoichiometry, and rows indicating the mass balance for each metabolite. With GECKO, this approach is expanded by adding new rows to the matrix to represent the enzymes and new columns to represent each enzyme usage. Kinetic information, in the form of *k*_cat_ values, is included as stoichiometric coefficients to convert the metabolic flux in mmol/gDWh to the required enzyme usage in mmol/gDW. The protein level is included as an upper bound for each enzyme usage, ensuring that the desired constraint on each flux is respected.

To create your own GECKO model, *k*_cat_ values of reactions, the molecular weight of proteins, and protein activity information will be required and can be directly changed and included in your GEM, because different metabolic groups have different *k*_cat_ values and molecular weight distribution [80]. All molecular and enzymatic parameters could be automatically retrieved from the BRENDA database [84] and/or the UNIPROT database [85].

The GECKO toolbox is dependent on MATLAB and other packages. There is an option in Python to work with GECKO models using the MEWpy package. Both require ecModels and normalized proteomics data.

#### 2.2.4. Strategies for Integrating Proteomics and Transcriptomics Data

The integration of proteomics and transcriptomics data has become a crucial part of modern Systems Biology research. The combination of these two omics data types can provide a more comprehensive understanding of biological systems. Here, we will elaborate on the three strategies for integrating proteomics and transcriptomics data.

One of the challenges in integrating transcriptomics and proteomics data is the difficulty in obtaining the same sets of differentially expressed genes and differentially expressed proteins. This is often due to differences in the timing of sample collection for RNA and protein analysis. RNA samples are typically collected at the transcription stage, whereas protein samples are collected at the translation stage. As a result, there can be significant differences in the expression patterns of genes and proteins between these two stages. Furthermore, RNA and protein stability can also differ, which can further complicate the comparison between these two types of data.

To overcome this challenge, it is important to carefully plan the experimental design and sample collection protocols. Ideally, the samples for both RNA and protein analysis should be collected at the same time point and under the same conditions. If this is not possible, researchers can try to account for the differences between RNA and protein data by using statistical methods to normalize the data or by applying machine learning algorithms to identify patterns of expression that are consistent across both data sets.

##### Differentially Expressed Genes and Proteins

The first strategy for integrating proteomics and transcriptomics data is Venn diagram analysis or Jaccard index calculation. In this strategy, differentially expressed genes (DEGs) and differentially abundant proteins (DAPs) are identified in the same biological conditions from both types of omics data. A Venn diagram is then used to identify overlapping genes and proteins, which can provide insights into the mechanisms underlying a certain biological condition. This strategy can be particularly useful for identifying key pathways or processes that are regulated by both proteins and transcripts. However, it is important to ensure that the transcription and translation processes are aligned at the time of collection to avoid false positives.

There are articles that show that there is not a great intersection between DEGs and DAPs [86,87,88,89,90,91,92,93,94,95], and others showing a successful intersection [96,97,98,99,100]. This discrepancy is based on the following: (i) induced and repressed proteins behaving differently, revealing regulatory and kinetic differences in protein synthesis and turnover; (ii) taking into account the transcription–translation delay when comparing protein and mRNA levels during dynamic adaptation; (iii) protein variation being mainly influenced by mRNA concentration in a new steady state [99]. Furthermore, if there is a significant overlap between DEGs and DAPs or if there is a GO/KEGG enrichment common to both, using the intersection list is the most appropriate approach. If there are many differences, the most common strategy is to construct a Venn diagram of the GO processes and KEGG pathways to identify the similarities and differences.

##### Observing Delays between Omics Data

The second strategy for integrating proteomics and transcriptomics data is scatter plot analysis. This strategy involves plotting the log of the genes fold change by the log of the proteins fold change. By observing the scatter plot, we can identify whether there is a correlation between the proteomics and the transcriptomics data. A positive correlation in the scatter plot suggests agreement in data extraction and provides a better understanding of the mechanisms underlying the biological condition. Scatter plot analysis can also be useful for identifying genes or proteins that do not have a direct correlation between their expression and protein levels based on the problems as cited before.

**Figure 2:**
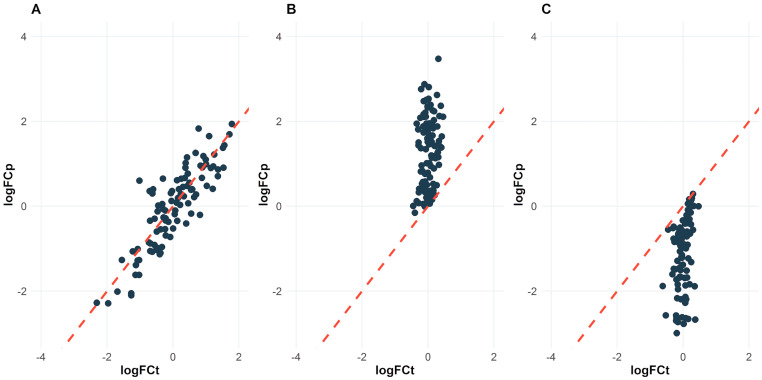
Scatter plot between the log fold change from a differential gene expression test (logFCt) and the log fold change of the protein levels (logFCp) in three scenarios: (**A**) high association between transcriptomics and proteomics data; (**B**,**C**) disagreement between the changes in gene expression and protein levels for some genes/proteins. The red dashed 45-degree line indicates the theoretical correspondence where changes in gene expression at the RNA-Seq level would be equally reflected at the protein level.

When we scatter plot the log fold change from a differential genes expression test (logFCt) versus the log fold change of the protein levels (logFCp), we can observe different patterns that can provide insights into the agreement or disagreement between the transcriptomics and the proteomics data. In this case, the logFCt and logFCp values increase or decrease together (Figure 2A), meaning that the genes and proteins are co-regulated in the biological system. This pattern suggests that there is a good agreement between the transcriptomics and the proteomics data and the protein abundance changes can be explained by changes in gene expression. If you see a concentration of points above (Figure 2B) and below (Figure 2C) the 45-degree line in the scatterplot of logFCt versus logFCp, it means that there is a disagreement between the changes in gene expression and protein levels for some genes/proteins. In other words, the gene expression and protein levels of these genes/proteins do not show a consistent pattern across the biological condition studied. This could be due to various factors such as post-transcriptional regulation, protein stability, or differences in the sampling and processing methods between the two omics (transcriptomics and proteomics) data sets.

The R package ReactomeGSA [101] includes a function named “plot_correlations()” which generates a comparative scatter plot of transcriptomics and proteomics data, allowing for a quick assessment of the similarity between the two data sets at the pathway level.

##### Interactome Analysis

The third strategy for integrating proteomics and transcriptomics data is interactome analysis. With this strategy, we could generate an interactome from differentially abundant proteins (DAPs) or differentially expressed genes (DEGs), identify submodules and hubs, and apply the fold change in gene expression to the interactome. This approach can help to identify functional relationships between different proteins and genes, providing a more comprehensive understanding of a biological system. Interactome analysis can also help to identify potential targets for further analysis, such as drugs or therapies.

First, from the list of DEGs, for example, we can generate an interactome using a protein–protein interaction (PPI) database, such as BioGRID [102] or STRING [103]. From the generated interactome, we can extract network metrics and identify hub genes and also submodules. With the application of the fold change in gene expression in the network, we can identify submodules with a predominance of a biological condition and apply biological enrichment to support such predominance.

Second, we can integrate this type of analysis based on co-expression analysis as well. Once the co-expression modules have been identified, it is possible to construct an interactome network that represents the interactions between the proteins encoded by these genes. The interactome can then be visualized and analyzed using network analysis tools such as Cytoscape, which allows for the identification of subnetworks, central nodes, and pathways that are enriched for the genes of interest, as well as of potential drug targets or biomarkers for diseases.

### 2.3. Machine Learning Methods Based on Omics Data

**Figure 3:**
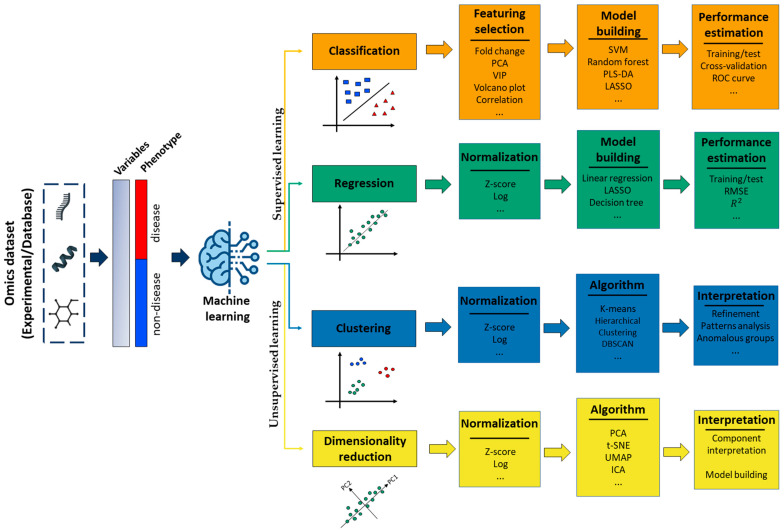
The pipeline illustrates the differences between supervised and unsupervised learning strategies applied to omics data. Legend: PCA: Principal Component Analysis; t-SNE: t-Distributed Stochastic Neighbor Embedding; UMAP: Uniform Manifold Approximation and Projection; ICA: Independent Component Analysis; SVM: Support Vector Machines; PLS-DA: Partial Least-Squares Discriminant Analysis; LASSO: Least Absolute Shrinkage and Selection Operator; RMSE: Root-Mean-Square Error; VIP: Variable Importance in Projection; ROC Curve: Receiver Operating Characteristic Curve.

In this section, we will explore various methods that utilize omics data such as transcriptomics, proteomics, and metabolomics data for supervised and unsupervised machine learning algorithms (Figure 3). Supervised learning methods involve machine learning algorithms that use known data–outcome pairs as examples, whereas unsupervised methods operate on data sets without an outcome variable or prior knowledge of relationships between observations, dealing with unlabeled data. These strategies employ different methodologies and can involve the use of one, two, or three types of omics data. Additionally, numerous studies and reviews have extended this discussion to cover other strategies and provide valuable tools to apply these methodologies with a greater emphasis on machine learning and multi-omics data [4,6,104,105,106,107,108].

#### 2.3.1. Transcriptomics Data

Gene expression data can provide insights into the complex interplay between genes and cellular processes. With the advent of high-throughput technologies such as microarrays and RNA sequencing, it is now possible to generate large-scale gene expression data sets for a wide range of biological systems. In recent years, machine learning algorithms have emerged as a powerful tool for analyzing gene expression data. By leveraging the computational power of machine learning, researchers can uncover complex patterns and relationships within data that would be difficult or impossible to detect using traditional statistical methods, such as unsupervised machine learning methods.

In the literature, numerous studies have demonstrated the successful application of classification methods to predict cancer and cell types by utilizing gene expression data from microarray or bulk RNA-Seq data [109,110,111,112,113,114,115] and single-cell transcriptomics [116,117,118,119,120,121,122]. The process of employing gene expression data commences with the selection of suitable data sets, which can be accessed from databases like GEO [123], TCGA [124], and SRA [124]. Subsequently, pre-processing is carried out on the corresponding metadata for the classification algorithms, such as data from patients with or without cancer. Following this, the data set may or may not undergo a feature selection process followed by the application of a sampling technique to reach the final classification model, but a cross-validation step is essential. Each of these steps uses different data sets, pre-processing steps, model training and prediction algorithms, along with different k-fold cross-validation values, leading to varying values of accuracy, sensitivity (measuring the proportion of true positives accurately identified), and specificity (measuring the proportion of true negatives accurately identified).

It should be noted that the accuracy of machine learning classifiers using RNA-Seq data is dependent on various factors such as the type of sequencer used, the library preparation method, and the sample preparation technique. As a result, these classifiers exhibit varying levels of accuracy, with better performance observed at the transcript level compared to the gene level [125].

Biomedical researchers need to confirm the biological significance of the list or cluster of genes associated with a particular condition or developmental process that have been identified through comprehensive data analysis. To achieve this, they must evaluate the false-positive rate and conduct an autonomous biological validation. Northern blots and PCR-based methods are commonly employed to verify gene expression data, and these methods have the advantages of being able to screen through a large number of candidates relatively rapidly and perform quantitative measurements. Additionally, in situ hybridization and immunohistochemistry are used to determine the precise tissue in which the candidate genes are expressed [126]. Although these methods are not typically quantitative or high-throughput, they can be used to screen a large number of candidate genes and, in some cases, be performed in a quantitative manner.

#### 2.3.2. Proteomics Data

With the advent of high-throughput technologies, such as mass spectrometry (MS) and protein microarrays, it is now possible to identify and quantify thousands of proteins in a single experiment. However, the sheer volume and complexity of proteomics data presents a challenge for traditional statistical and computational methods. Machine learning algorithms offer a promising solution for analyzing and interpreting these data, enabling researchers to extract meaningful information about protein function, interactions, and disease mechanisms. In this section, we will discuss how machine learning algorithms can be applied to proteomics data, including feature selection, classification, and clustering methods.

According to the literature, the machine learning algorithms used for proteomics data are based on retention time prediction, MS/MS spectrum prediction, the identification of peptides, biomarker identification, bias reduction during data processing, secondary structure prediction, protein toxicity prediction, protein function, and protein interactions [127,128,129,130,131,132,133,134,135,136,137,138,139,140,141,142]. Considering a comprehensive and reliable data integration, processing proteomics data and utilizing machine learning algorithms to accurately identify and analyze proteins require a consolidated pipeline. Due to the complexity of proteomics data, a comprehensive approach to data processing, normalization, quality control, feature extraction, and statistical analysis is necessary [7]. Without a consolidated pipeline, the analysis of proteomics data can be prone to errors and inconsistencies, leading to inaccurate results.

Identifying a biomarker from machine learning models based on proteomics data poses several challenges, including the need for validation in independent tests and the demonstration of clinical utility. These challenges are crucial in the translation of a promising biomarker candidate into a clinical tool. Validating the findings in independent tests is essential to ensure that the identified biomarker is reliable and reproducible. Demonstrating its clinical utility is also necessary to prove that the biomarker can effectively diagnose, monitor, or predict disease outcomes. These challenges highlight the importance of rigorous testing and validation before the implementation of any biomarker-based clinical assay.

#### 2.3.3. Metabolomics Data

Machine learning has been applied to metabolomics data to identify biomarkers associated with diseases, understand metabolic pathways, contribute to the development of biotechnologies, and predict drug responses. PCA is one of the most widely used techniques to analyze metabolomics data, where metabolites are reduced into principal components that represent the majority of the variability in the data. PCA can identify metabolites that are significantly associated with different biological conditions, such as healthy and disease states, and can be used to cluster samples based on their metabolic profiles [143,144,145,146,147,148].

The variable in projection (VIP) score from the partial least-squares–discriminant analysis (PLS-DA) is another important method in metabolomics data analysis that can be used to identify relevant metabolites associated with a particular biological condition [149,150,151,152,153,154,155]. The VIP score is a measure of the contribution of each metabolite to the separation between two groups and is calculated by applying a supervised learning algorithm to the data, such as the PLS-DA algorithm. The MetaboAnalyst online platform is an example of a tool that allows researchers to apply VIP score analysis to their metabolomics data, as well as to perform other statistical and machine learning analyses.

Metabolomics data have been widely used in machine learning models to predict different types of cancer as well, as they provide valuable information about metabolic pathways altered in cancer cells. For instance, recent studies used metabolomics data in combination with machine learning algorithms to distinguish between different types of cancer, including lung, breast, non-Hodgkin’s lymphoma, and ovarian cancer, and non-cancer conditions, such as coronavirus disease (COVID-19), type-2 diabetes, acute myocardial ischemia, schizophrenia, and autism in relation to gestational age [156]. Moreover, the identification of biomarkers in several of these cited studies has the potential to improve disease diagnosis, treatment, and monitoring. It allows for the discovery of complex relationships between metabolites and biological conditions that may not be easily detected through traditional methods.

Transcriptomic data are still used to predict metabolite concentrations using machine learning models. Auslander N. et al. (2016) [157] demonstrated that the levels of a wide range of metabolites in breast cancer can be successfully predicted from the transcriptome. The authors developed a Support Vector Machine (SVM) classifier to identify reaction-gene–metabolite (RGM) triplets where the gene and metabolite involved in the same reaction showed a significant association, whether positive or negative.

Selecting the suitable ML algorithm (including linear regression, logistic regression, support vector machines, k-nearest neighbors, decision trees, random forests, neural networks, and deep learning) plays a critical role in the achievement of a metabolomics study. It is crucial for researchers to be knowledgeable about the advantages of various ML approaches and to choose the most appropriate one based on their requirements to obtain accurate and easily understandable results [156,158].

## 3. Conclusions

In conclusion, integrating multiple omics data types is a powerful approach that can provide a more comprehensive understanding of biological systems. Transcriptomics, proteomics, and metabolomics offer complementary views of biological processes at the RNA, protein, and metabolite levels, respectively. By combining these data types, researchers can gain insights into complex biological phenomena that may not be possible with any single omics data type alone.

There are several strategies for integrating omics data, including co-expression analysis, pathway analysis, and network analysis. Each strategy has its strengths and weaknesses, and the choice of the approach will depend on the specific research question being addressed.

Overall, the integration of omics data is a rapidly evolving field, with new methods and tools being developed to address the challenges of analyzing and interpreting large and complex data sets. As technology continues to advance, the integration of omics data is likely to become even more important for understanding molecular mechanisms in biology.

In this article, we reviewed several methods for integrating omics data and provided examples of their application in various biological contexts. Moreover, we explored the applications of omics data in machine learning studies. We hope that this review will inspire further research in this field and lead to new insights into the complex interplay between genes, proteins, and metabolites in living systems.

## Figures and Tables

**Table 1:** Summary of methods and strategies for integrating transcriptomics, proteomics, and metabolomics data using the correlation-based approach.

Integration Approach	Strategy or Method	Possible Omics Data	Main Idea
Correlation-based	Gene co-expression analysis	Transcriptomics and metabolomics	Identify co-expressed gene modules with metabolite similarity patterns under the same biological conditions
	Gene–metabolite network	Transcriptomics and metabolomics	Perform a correlation network of genes and metabolites
	Similarity Network Fusion	Transcriptomics, proteomics, and metabolomics	Builds a similarity network for each omics data separately, and subsequently, all networks are merged, and the edges with high associations in each omics network are highlighted
	Enzyme and metabolite-based network	Proteomics and metabolomics	Identify a network of protein–metabolite or enzyme–metabolite interactions using genome-scale models or pathways databases

**Table 2:** Summary of methods and strategies for integrating transcriptomics, proteomics, and metabolomics data using the combined omics approach.

Integration Approach	Strategy or Method	Possible Omics Data	Main Idea
Combined omics	Pathway enrichment from differentially expressed genes and metabolites	Transcriptomics and metabolomics	Identify pathways enriched in both types of omics data and perform a post-analysis with these results
	Integrating genome-scale models with omics data	Transcriptomics and metabolomics	Integrate metabolic and transcriptomic data to create content-specific models and perform specific metabolic simulations
	Gecko models	Proteomics and metabolomics	Integrate proteomics data into an enzyme model, which can be validated with metabolomics data under the same biological conditions.
	Differentially expressed genes and proteins	Transcriptomics and proteomics	Identify similarities between the lists of differentials in the two omics data sets
	Observing delays between omics data	Transcriptomics and proteomics	Identify whether there is a temporal delay in the acquisition of omics data based on gene expression and protein abundance
	Interactome analysis	Transcriptomics and proteomics	Identify functional relationships between different proteins and genes using interactome databases and fold-change values

## Data Availability

No new data were created or analyzed in this study. Data sharing is not applicable to this article.
